# Loss of the maternal effect gene *NLRP2* impairs embryonic and extra-embryonic development, revealing a novel genetic cause of congenital anomalies^†^

**DOI:** 10.1093/biolre/ioaf290

**Published:** 2025-12-27

**Authors:** Momal Sharif, Zahra Anvar, Imen Chakchouk, Sara H El-Dessouky, Roni Zemet, Eric C Kao, Wessam E Sharaf-Eldin, Ying-Wooi Wan, Zhandong Liu, Pengfei Liu, Michael Jochum, Ignatia B Van den Veyver

**Affiliations:** Department of Obstetrics and Gynecology, Baylor College of Medicine, Houston, TX, USA; Duncan Neurological Research Institute at Texas Children’s Hospital, Houston, TX, USA; Department of Obstetrics and Gynecology, Baylor College of Medicine, Houston, TX, USA; Duncan Neurological Research Institute at Texas Children’s Hospital, Houston, TX, USA; Department of Obstetrics and Gynecology, Baylor College of Medicine, Houston, TX, USA; Duncan Neurological Research Institute at Texas Children’s Hospital, Houston, TX, USA; Prenatal Diagnosis & Fetal Medicine Department, National Research Centre, Cairo, Egypt; Department of Prenatal Diagnosis and Fetal Medicine, Human Genetics and Genome Research Institute, National Research Centre, Cairo, Egypt; Department of Obstetrics and Gynecology, Baylor College of Medicine, Houston, TX, USA; Department of Molecular and Human Genetics, Baylor College of Medicine, Houston, TX, USA; Baylor Genetics Laboratories, Houston, TX, USA; Department of Prenatal Diagnosis and Fetal Medicine, Human Genetics and Genome Research Institute, National Research Centre, Cairo, Egypt; Medical & Molecular Genetics Department, National Research Centre, Cairo, Egypt; Duncan Neurological Research Institute at Texas Children’s Hospital, Houston, TX, USA; Department of Pediatrics-Neurology, Baylor College of Medicine, Houston, TX, USA; Duncan Neurological Research Institute at Texas Children’s Hospital, Houston, TX, USA; Department of Pediatrics-Neurology, Baylor College of Medicine, Houston, TX, USA; Department of Molecular and Human Genetics, Baylor College of Medicine, Houston, TX, USA; Baylor Genetics Laboratories, Houston, TX, USA; Department of Obstetrics and Gynecology, Baylor College of Medicine, Houston, TX, USA; Department of Obstetrics and Gynecology, Baylor College of Medicine, Houston, TX, USA; Duncan Neurological Research Institute at Texas Children’s Hospital, Houston, TX, USA; Department of Molecular and Human Genetics, Baylor College of Medicine, Houston, TX, USA

**Keywords:** maternal effect genes, Nlrp2, embryonic development, congenital anomalies, pregnancy loss, imprinting disorders

## Abstract

Maternal effect genes (MEGs) play a crucial role in early mammalian development, and their dysfunction can lead to severe embryonic and extra-embryonic abnormalities. *NLRP2*, a MEG that encodes a subcortical maternal complex protein, has been implicated in preimplantation development, but its role after implantation remains underexplored. In this study, we investigated the developmental consequences of maternal *Nlrp2* loss-of-function in a maternal knockout (KO) mouse model at embryonic day 11.5. Embryos derived from *Nlrp2*-KO females have abnormal yolk sac vasculature, increased embryonic resorption, craniofacial abnormalities, neural tube defects, and congenital heart defects. Placental architecture is disrupted with an altered junctional zone and labyrinth structure. Transcriptome profiling of maternal decidua and placenta demonstrated dysregulation of genes involved in trophoblast differentiation and extra-embryonic development. Bisulfite sequencing of these tissues revealed persistence at E11.5 of previously observed locus-specific disruption in DNA methylation at four imprinted loci following maternal *Nlrp2* loss. We further describe pregnancy outcomes and offspring phenotypes for two unrelated women with bi-allelic maternal *NLRP2* variants. The first carried homozygous *NLRP2* nonsense variants and experienced recurrent pregnancy loss and fetuses with multiple structural anomalies, including omphalocele, craniofacial dysmorphism, and cardiac defects. The second carried compound heterozygous frameshift and missense *NLRP2* variants and had a child with neurodevelopmental impairment of uncertain etiology. These findings indicate a conserved role for maternal NLRP2 in embryonic viability and placental development, and support further studies in humans into the contribution of *NLRP2* and other similar MEGs to offspring congenital anomalies and adverse pregnancy outcomes.

## Teaser

Maternal *NLRP2* loss of function disrupts embryonic and placental development, leading to congenital anomalies and adverse reproductive outcomes in both mice and humans.

## Graphical Abstract

**Graphical Abstract:**
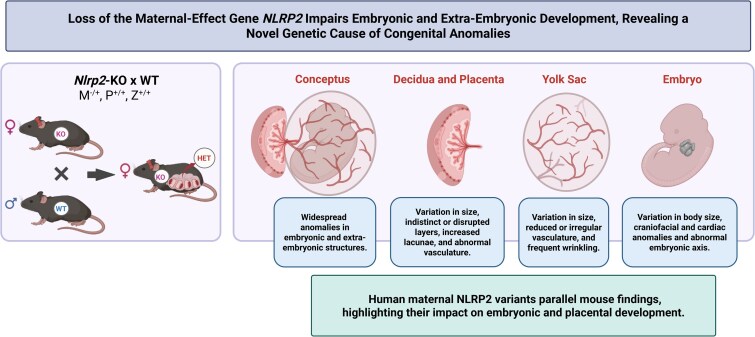

## Introduction

A subset of women with unexplained clinical infertility have non-diagnostic results with standard infertility work-up and produce oocytes that can be fertilized but fail to develop beyond the cleavage or blastocyst stage. Others may experience pregnancies that can progress further but result in recurrent pregnancy loss or live births with developmental disabilities linked to disrupted imprinting [1–5]. Some women have loss-of-function variants in maternal effect genes (MEGs). Proteins encoded by MEGs accumulate during oocyte development and have essential roles in early embryonic development, including epigenetic regulation and cytoplasmic organization [2, 6, 7]. These proteins also contribute to maternal-to-zygotic transition (MZT) and zygotic genome activation (ZGA), which marks the shift in transcription from the maternal genome to the embryonic genome at the 2-cell stage in mice and the 8-cell stage in humans [6, 8].

Human and mouse oocytes and early embryos contain a set of maternal-effect proteins, known as subcortical maternal complex (SCMC) proteins [1, 9–11]. Recent studies show that SCMC proteins are present throughout the ooplasm and remain closely associated with the oocyte during the oocyte-to-embryo transition [12]. They regulate multiple processes, including mitochondrial distribution, cytoplasmic lattice formation, cortical granule release, spindle positioning, cytoskeletal organization, genome stability, and activation of the embryonic genome [1, 6, 9–14]. Disruption or inactivation of genes that encode SCMC proteins hinders early embryonic development and epigenetic reprogramming in part by disrupting nuclear and cytoplasmic structures [6, 10, 15, 16]. In humans, recessive loss-of-function variants in several SCMC genes cause reproductive disease, including hydatidiform moles, recurrent pregnancy loss, and imprinting disorders in offspring [6, 17–21].

**Figure 1:**
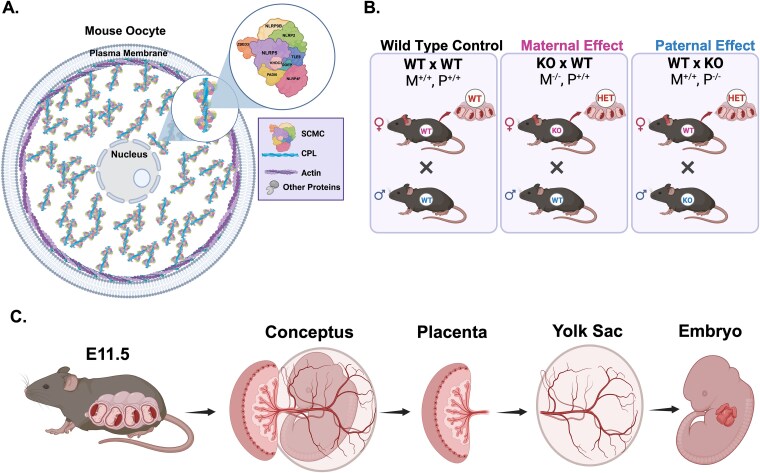
Characterization of maternal loss of *Nlrp2*. (A) Diagram depicting the mouse oocyte with cytoplasmic lattices (CPL) and the SCMC structure. (B) Mating schemes used for the comprehensive analysis of concepti (embryos within the yolk sac and attached placentas). Three mating crosses were established: wildtype control (WT x WT), maternal effect (KO x WT), and paternal effect (WT x KO). (C) Schematic of the timed pregnancy at embryonic day 11.5 (E11.5). The concepti collected were used for histology, μCT analysis, transcriptomic profiling, and DNA methylation studies.

Core SCMC proteins in mice include the NOD-like receptor family pyrin domain-containing 5 protein (NLRP5/ MATER), oocyte-expressed protein Oocyte Expressed Protein (OOEP/ FLOPED), and Transducin-like enhancer protein 6 (TLE6), which form a stabilizing platform for the complex [12]. Additional proteins in the complex include KH Domain Containing 3 (KHDC3 /FILIA), and NOD-like receptor family pyrin domain containing proteins (NLRP4F, NLRP9B, and NLRP2) (Figure 1A). In humans, NLRP7 and KHDC3L (ortholog of KHDC3) are also a part of the complex [12, 22]. Many SCMC-associated proteins belong to the NLR family. These proteins were initially recognized for their immune functions but are now known to support reproductive processes [12]. Most human NLRPs have orthologs in mice, except for NLRP7, which is most closely related to mouse NLRP2. In humans, maternal recessive pathogenic variants in NLRP2, NLRP5, and NLRP7 cause multilocus imprinting disorders characterized by DNA methylation defects at multiple imprinted loci [11, 23, 24]. NLRP7 variants cause biparental hydatidiform moles, while maternal NLRP2 variants are associated with offspring imprinting disorders, such as Beckwith–Wiedemann syndrome, featuring variable fetal and neonatal overgrowth, facial dysmorphism, and organomegaly [2, 6, 24–27]. Recent studies have further implicated NLRP2 variants in defective SCMC function and oocyte-driven embryogenesis [28]. The increasing recognition of NLRP2 variants in early embryonic arrest and infertility [2, 3, 28–30] suggests that this gene plays a critical role in early embryonic survival and extra-embryonic tissue formation.

We previously reported that maternal, but not paternal, loss of *Nlrp2* in mice results in a variably expressive reproductive phenotype that includes pre- and post-implantation developmental defects, some embryos capable of progressing to term, and liveborn and stillborn offspring [13, 17]. We also identified DNA methylation abnormalities at imprinted regions, including *Impact* and *Zac1*, in stillborn offspring from *Nlrp2*-deficient females, suggesting that maternal *Nlrp2* contributes to proper maintenance of imprinting [13, 17]. Our recent oocyte transcriptome studies revealed that maternal *Nlrp2* deficiency alters the expression of key epigenetic regulators and disrupts MZT, further supporting its role in early developmental programming [31]. However, the spectrum of congenital anomalies resulting from maternal *Nlrp2* loss and the pathways disrupted during post-implantation development have not been comprehensively examined.

Here, we used high-resolution microcomputed tomography (MicroCT; μCT) together with placental and maternal decidua (MD) transcriptome profiling to characterize embryonic and extra-embryonic defects in pregnancies of *Nlrp2*-KO females. We focused in particular on congenital heart defects (CHDs), abnormal yolk sac and MD vascularization, and placental layer defects, as their concurrent disruption may reflect shared developmental pathways [32–35]. To establish clinical relevance, we also present two unrelated women with maternal *NLRP2* variants: one with homozygous pathogenic nonsense variants who experienced recurrent pregnancy loss and pregnancies with multiple structural fetal anomalies, and a second who is a compound heterozygote for a frameshift and a missense variant who had a child with neurodevelopmental impairment of uncertain etiology. These mouse and human findings support the hypothesis that maternal loss of *Nlrp2* disrupts embryonic and placental development, leading to a range of abnormalities that parallel those observed in human pregnancies with maternal NLRP2 variants.

## Materials and methods

### Ethics statement

All animal experiments were performed in accordance with institutional and national guidelines for the care and use of laboratory animals. Mouse studies were approved by the Baylor College of Medicine Institutional Animal Care and Use Committee (Protocol Number: AN-2035). The animal facilities were accredited by the Association for Assessment and Accreditation of Laboratory Animal Care International. The prospective clinical study was conducted in accordance with the ethical standards of the National Research Centre, Cairo, Egypt, and approved by the Medical Research Ethical Committee of the National Research Centre (IRB Protocol Number: 21125062022). Written informed consent was obtained from the patient for all clinical evaluations, genetic testing, and the use of anonymized clinical data for research and publication. The retrospective clinical analyses were conducted in accordance with the ethical standards of Baylor College of Medicine and Baylor Genetics and approved by the Baylor College of Medicine Institutional Review Board (IRB Protocol Number: H-41191).

### *Nlrp2* mice

The validated *Nlrp2*-deficient mouse model *Nlrp2*^*t*m1a/tm1a^ (Null or KO) was previously generated on a C57BL6/J background and genotyped as described [13]. To generate *Nlrp2^+/+^* (WT) and *Nlrp2*^tm1a/tm1a^ (KO) male and female mice for experiments, we bred *Nlrp2*^+/tm1a^ (HET) females *Nlrp2*^+/tm1a^ (HET) males (both ≥8 weeks old). We weaned pups on postnatal day 21 and separated and group-housed female and male pups with up to four animals per cage and provided ad libitum access to standard water, feed (Pico Lab, LabDiet), and nesting material. After genotyping, we selected WT and KO animals for all further experiments. We set up 5–14 timed matings each for the following mating crosses: wildtype control (WT x WT), maternal effect (KO x WT), and paternal effect (WT x KO) (Figure 1B). We counted the presence of a vaginal plug as embryonic day (E) 0.5 and humanely euthanized pregnant females at E11.5 to collect, examine, and process tissues for experiments (Figure 1C). Embryos and placentas were either fixed in 4% paraformaldehyde (PFA) overnight at 4°C or snap-frozen in liquid nitrogen and stored at −80°C for further processing based on experimental design.

### Histology

We dissected conceptuses in 1X phosphate-buffered saline (PBS) and fixed them with 4% PFA (Sigma) overnight at 4°C. We also bisected the placentas along the midline and fixed them in 4% PFA for histological studies. We washed and fixed tissues twice for 45 min in 1X PBS and subsequently dehydrated them for 1 h in 50% ethanol and 1 h in 70% ethanol, and three times for 1 h each in 100% ethanol at room temperature (RT). We then treated tissues three times for 30 min each with xylene, and twice for 1 h each in paraffin changes at 55°C before paraffin embedding. We serially sectioned paraffin-embedded tissues at 5–8 μm thicknesses and stained them with hematoxylin and eosin (H&E). We identified sections through the placental midline based on the insertion of the umbilical cord and analyzed at least five sections, positioned 60–70 μm apart from each side of the placental midline for assessment of placental layers.

### MicroCT analysis

After euthanizing pregnant dams, we carefully excised the uterine horns containing intact conceptuses and placed the dissected horns in warm PBS and then fixed them by immersion in 4% PFA (Sigma) at 4°C overnight. Following fixation, we rinsed the samples three times in 1X PBS before immersing them overnight in a 0.1 N (v/v) iodine solution (Sigma) at RT. The following day, we embedded each sample individually in 400 μl of 1% w/v agarose in a 2 ml-screw-cap microcentrifuge tube (VWR) and promptly imaged them. We used a SKYSCAN 1272 Micro-CT scanner (Bruker) to generate the raw data for 3D imaging of the samples. Following this, we reconstructed the obtained projection images using the NRecon Reconstruction software (Bruker) and visualized the reconstructed 3D data for each sample to render the entire volume in 3D using CTVox (Bruker). We used IMARIS software (Oxford Instruments) to perform a comprehensive 3D segmentation of the conceptuses. All μCT image analysis, segmentation, and phenotypic scoring were performed blinded to genotype until completion of quantitative assessments. Additional imaging parameters, acquisition settings, and reconstruction procedures are provided in the Supplemental Methods.

### Maternal decidua and placental RNA extraction

We extracted total RNA and DNA from snap-frozen tissue units consisting of both MD and whole placenta from Maternal *Nlrp2*-KO matings and WT control matings (*n* = 4 for each genotype combination, all from different mating pairs). Gross morphological characteristics were documented before freezing**.** We used the miRNeasy Mini Kit (Qiagen) following the manufacturer’s instructions for RNA extraction. RNA and DNA were extracted from the same tissue aliquots.

### RNA sequencing and library preparation

RNA integrity and concentration were assessed using spectrophotometric, fluorometric, and electrophoretic quality-control assays. RNA-seq library preparation and sequencing were performed at the Baylor College of Medicine Genomic & RNA Profiling Core using the NEBNext Ultra II Directional RNA library preparation workflow with rRNA depletion. Libraries were indexed, quantified, pooled equimolarly, and sequenced on an Illumina NovaSeq 6000 platform using paired-end 150 bp reads. An average sequencing depth of approximately 100 million read pairs per sample was obtained. Detailed procedural steps, reagent specifications, and quality-control parameters are provided in the Supplemental Methods.

### RNA sequence data analysis

We first checked raw reads for quality using FastQC [36], trimmed off the adapters using awk, and aligned trimmed reads to the reference mouse genome (GRCm39_vM27) using STAR v2.6.0a [37]. We processed primary alignment BAM files sorted by coordinates using RSeQC, which we found to be uniform for all samples [38]. We combined output reports from FASTQC, STAR, and RSeqQC into a multireport using multiQC [39]. A tab-delimited matrix consisting of reads per gene for each sample and a sample metadata sheet was then imported in R [40], merged, and converted into a DESeqDataSet class using DESeq2 [41]. We applied a regularized transformation for plotting the MA and principal component analysis (PCA) plots and correlation heatmaps [40]. Principal component analysis, sample correlation assessment, and visualization of differentially expressed genes (DEGs) were performed using standard workflows within R [40]. Full details of the bioinformatic pipeline, software versions, and quality-control parameters are provided in the Supplemental Methods.

### Maternal decidua and placental layer marker heatmap methods.

Differentially expressed genes derived from DESeq2 analysis, and the corresponding count matrix were filtered by gene name for predefined cell marker gene subgroups 1, 2, 3, and 4, as defined in Supplemental Files 1–2. These subsets were organized into individual data frames for downstream data visualization using ggpubr and ComplexHeatmap [42]. Marker genes for each heatmap were clustered based on Euclidean distance using Ward D2 clustering, and columns were annotated for base means, log_2_FoldChange, *P*-value, and mean counts for each genotype. After annotation and clustering, a final combined complex heatmap matrix was generated, with ordering along the y-axis based on genotype across all marker-based gene groups.

### Estimating cell composition based on marker gene structure

To estimate cell composition across maternal decidual and placental layers, DEGs derived from DESeq2 analysis were filtered based on predefined cell marker gene groups (Supplemental Files 1–2). The resulting marker gene count matrix was used for decomposition analysis. Marker-based decomposition was performed using the BisqueRNA package [43], generating bulk proportion estimates of the relative abundances of different cell types from PCA. A list of principal component values explaining the variance for each cell marker group was also generated.

### Quantitative reverse transcription PCR (RT-qPCRs)

Previously extracted RNA (500 ng) isolated from the MD and whole placenta was reverse transcribed into cDNA using the qScript cDNA SuperMix (Quanta Biosciences). Quantitative RT-PCR of reverse-transcribed cDNAs was done with PerfeCTa® SYBR® Green FastMix ROX (Quanta Biosciences) using the primers listed in the Supplemental File 3 on a real-time PCR thermocycler (Bio-Rad). Gene expression analyses were performed using the ΔΔCt method and normalized against the housekeeping gene *Gapdh* and are displayed as mean relative to the WT control samples. Additional experimental details and statistical parameters are provided in the Supplemental Methods.

### Bisulfite sequencing

Genomic DNA was isolated from placental tissue (*n* = 4 per group) from maternal *Nlrp2*-KO and WT control pregnancies. Bisulfite conversion was performed using the EZ DNA Methylation-Direct Kit (Zymo Research). Selected regions in four imprinting-associated genes (*Zac1, Mest, Ascl2*, and *Zfp64*) were amplified with locus-specific primers, cloned, and sequenced for methylation analysis. For each amplified locus, 10 colonies per biological replicate were sequenced and analyzed using Quantification Tool for Methylation Analysis [44] to quantify CpG methylation, and low-quality sequences were excluded using standard conversion and alignment thresholds.

Percent methylation was calculated per placenta by averaging CpG methylation across all informative clones for each locus. The placenta was treated as the independent biological unit for statistical analysis; individual colonies and CpG sites were considered technical measurements and were not treated as independent samples. Group comparisons were performed using an unpaired two-tailed *t-*test on placental mean percent methylation values, with significance defined as *P* < 0.05. Full experimental details, including primer sequences, cloning workflow, conversion criteria, and analysis parameters, are provided in the Supplemental File 4 and Supplemental Methods.

### Clinical studies and human genetic analysis

To explore the clinical relevance of maternal NLRP2 dysfunction observed in the mouse model, we identified and studied two independent human mother-offspring dyads. The first was identified at the National Research Centre, Cairo, Egypt, because of current and prior pregnancies and offspring with multiple fetal anomalies and a normal karyotype (Clinical presentation is described in detail in the Results and Supplemental File 5). Exome sequencing was performed on parental blood samples, and variants were analyzed to identify rare pathogenic alterations in MEGs. The second case was identified from a retrospective review of genome-wide exome analyses from 3000 prenatal and postnatal proband-parent trios evaluated at Baylor Genetics and Baylor College of Medicine. As a part of this study, we aimed to identify associations between congenital anomalies that were unexplained after analysis focused on the fetal genome and MEG variants in the maternal genome. A curated set of 113 MEGs was compiled from prior literature, and 42 were prioritized based on their known roles in early development (Supplemental File 6), imprinting disorders, or structural anomalies. Maternal variants were screened for rare, potentially pathogenic variants in these genes, and phasing was assessed by trio segregation. Variant interpretation for both cases followed ACMG guidelines [45]. Additional sequencing methods, bioinformatic pipelines, and variant filtering procedures are provided in the Supplemental Methods.

### Data analysis

All plotted data were represented as mean ± standard error (SEM), and *P* < 0.05 was used as the significance cut-off for normally distributed data. For continuous variables, statistical significance was assessed using one-way ANOVA with appropriate post hoc tests to account for unequal variances. Categorical or frequency-based comparisons were evaluated using Fisher exact test. All data graphs were generated using GraphPad Prism software version 10.0.2.

## Results

To comprehensively characterize the impact of the maternal loss of function of *Nlrp2* in mice, we established three mating schemes (Figure 1B) between the previously validated *Nlrp2-*Knockout (KO) mice [13] and wild-type (WT) mice from the same genetic background. These were designated as “Maternal Effect” (KO female x WT male), “Paternal Effect” (WT female x KO male), and “Control” (WT female × WT male) matings to distinguish the reproductive consequences of maternal *Nlrp2* loss from any potential effects of losing normal early post-implantation expression of the paternally contributed allele. We conducted a detailed phenotypic analysis at embryonic day 11.5 (E11.5), examining the entire conceptus, including yolk sacs, placentas, and embryos (Figure 1C). For some experiments, we primarily focused on the maternal effect and control mating schemes, because our observations and previously published findings [13, 17] found no significant impact of paternal loss-of-function of *Nlrp2* on reproductive phenotypes and outcomes.

### Maternal loss of *Nlrp2* causes developmental anomalies in concepti

**Figure 2:**
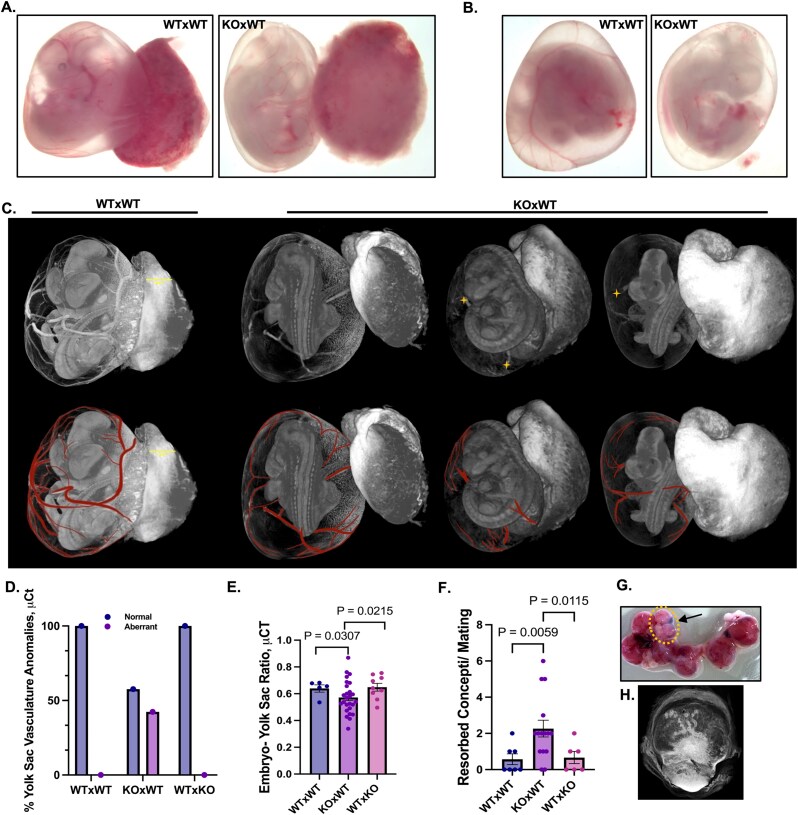
Maternal loss of *Nlrp2* causes developmental anomalies in concepti. (A–B) Microscopic Assessment of the entire E11.5 conceptus (embryo within the yolk sac and attached placenta), revealing paler embryos and yolk sacs from *Nlrp2-*KO dams compared to WT controls. (C) μCT images depict various anomalies in concepti from *Nlrp2-*KO dams (top panels). Pseudocolored yolk sac vasculature highlights differences between genotypes (bottom panels). Yellow “+” denotes the sites where wrinkles in the yolk sac were observed. (D) Percentage of concepti from *Nlrp2*-KO dams with aberrant yolk sac vasculature (*n* = 29, concepti from independent pregnancies). (E) Quantification of yolk sac to embryo area ratios indicates variable differences in the amniotic cavity (*n* = 29, concepti from independent pregnancies). (F) Increased frequency of resorbed embryos at E11.5 in *Nlrp2*-KO dams compared to other mating schemes (*n* = 15, pregnancies). (G) Uteri with concepti showing areas with resorbed embryos (dotted yellow circle). (H) μCT image of one of the resorbed embryos.

We conducted a comprehensive analysis of individual conceptuses as a unit, consisting of the embryo inside the yolk sac containing amniotic fluid, with the placenta still attached. We first performed microscopy imaging (Figure 2A and B) and found that embryos and yolk sacs from maternal effect (KO × WT) matings exhibited visibly reduced vasculature with variation in the sizes of concepti and were paler compared to those of control (WT × WT) matings. We also used X-ray microcomputed tomography (μCT) imaging for a more comprehensive three-dimensional (3D) evaluation of the entire concepti. We found a range of abnormalities in concepti of KO × WT matings, including abnormally sized and wrinkled yolk sacs (denoted by yellow **+** in Figure 2C), with evidence of reduced and abnormally positioned blood vessels (Figure 2C and D). To better illustrate vascular defects, yolk sac vessels were pseudocolored during image post-processing (Figure 2C, lower panels). Quantification showed that 42.4% of KO × WT conceptuses displayed aberrant yolk sac vasculature, whereas none were observed in WT × WT or WT × KO matings (Figure 2D). We also measured embryo to yolk sac area ratios and found that they were more variable and significantly different in concepti of KO × WT matings compared to the other two mating types (*P* = 0.0307; *P* = 0.0215; one-way ANOVA with multiple comparisons) (Figure 2E and Supplemental Figure 1A). There were also significantly more resorbed concepti in KO × WT matings compared to the other mating schemes (*P* = 0.0059; *P* = 0.0115; one-way ANOVA with multiple comparisons) (Figure 2F–H).

### Maternal loss of *Nlrp2* causes diverse embryonic anomalies, including heart defects

**Figure 3:**
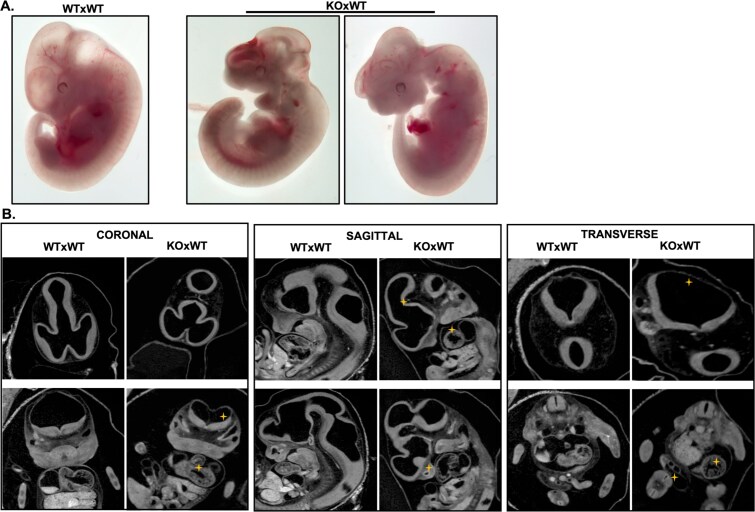
Maternal loss of *Nlrp2* causes developmental anomalies in embryos. (A) E11.5 embryos from *Nlrp2*-KO dams were examined using light microscopy and μCT. (B) Representative coronal, sagittal, and transverse μCT sections from WT × WT and KO × WT embryos. Embryos from KO × WT matings exhibited developmental anomalies (Yellow “+”), including altered midbrain morphology (sagittal), distorted neural tube architecture (coronal), and irregular outflow tract configuration (transverse). Craniofacial disorganization and overall asymmetry were also more pronounced in KO × WT embryos (*n* = 29, embryos analyzed by μCT from independent pregnancies). Detailed methodology for this analysis using emouseatlas and μCT is illustrated in the Supplemental Figure 1 and Videos 1–2.

**Figure 4:**
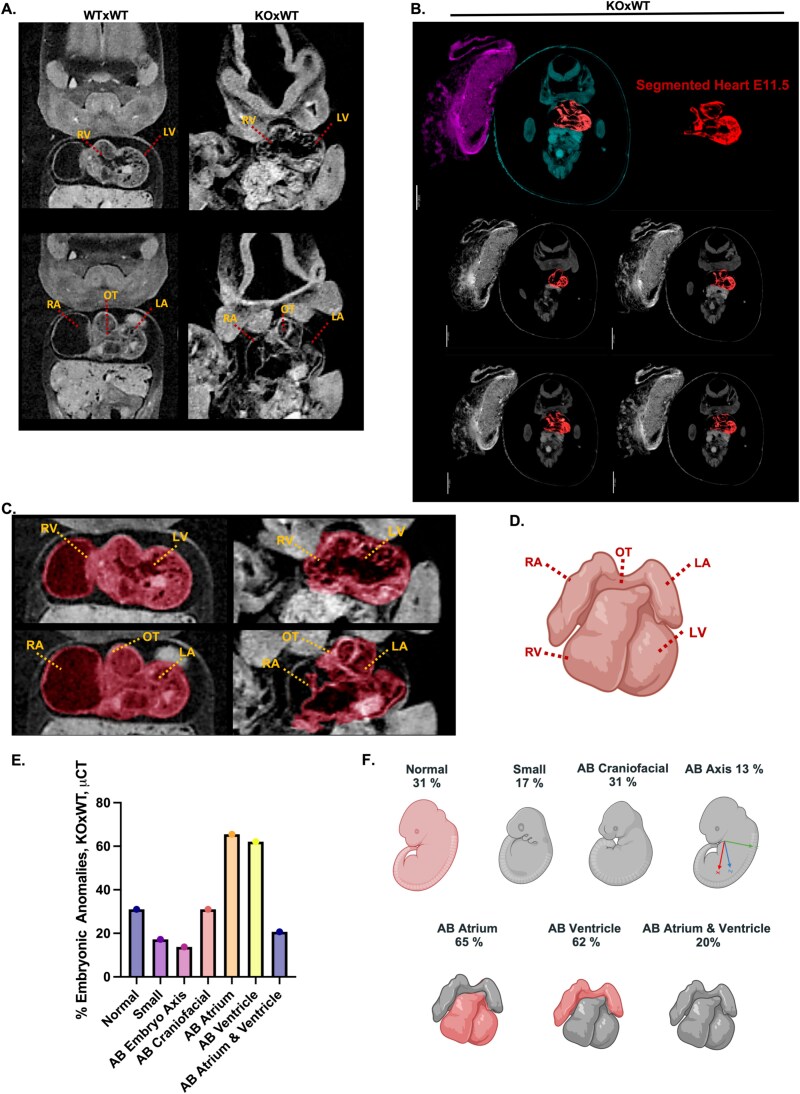
Maternal loss of *Nlrp2* causes congenital anomalies, including heart defects. (A) Representative 2D sectioning focused on the heart at E11.5 using μCT datasets to comprehensively assess congenital anomalies. (B–C) 3D segmentation of embryonic heart, pseudocolored to highlight structural anomalies. (D) Schematic representation of mouse heart structure (AB: Abnormal; RV: Right Ventricle; LV: Left Ventricle; OT: Outflow Tract; RA: Right Atrium; LA: Left Atrium). (E–F) Summary of spectrum of embryonic anomalies observed in E11.5 embryos from *Nlrp2*-KO dams: 31% normal, 17% smaller size, 31% craniofacial abnormalities, 13% abnormal embryonic axes, 65% abnormal atria, 62% abnormal ventricles, and 20% exhibited both atrial and ventricular abnormalities (*n* = 29, embryos analyzed by μCT segmentation from independent pregnancies). Detailed μCT image analysis and segmentation methodology are provided in Supplemental Figure 1 and Videos 1–3.

We next examined the embryos in detail by microscopy and μCT (Figure 3). Basic microscopy revealed that embryos from maternal effect (KO × WT) have variably altered body sizes and craniofacial anomalies (Figure 3A). We then measured the head, frontal-nasal, body, forelimb, hindlimb, and eyespot areas (Supplemental Figure 1B). None of these measurements were significantly different at *P* = 0.05 (one-way ANOVA with multiple comparisons) between embryos from maternal effect (KO × WT), control (WT × WT), and paternal effect (WT × KO) matings (Supplemental Figure 1C). To thoroughly characterize and classify the embryonic developmental defects, we compared standard cranial, sagittal, and transverse 2D sections of μCT-generated 3D embryo images to similar reference sections of wild-type embryos available in the resources of Kaufman Atlas of Mouse Development and eMouseAtlas [46] (Supplemental Figure 1D; Figure 3B). We utilized 3D-rendering and 2D-sectioning techniques (Supplemental Videos 1–2) and applied the basic histology template generated using mouse atlas images [46] shown in (Supplemental Figure 1D), which facilitated a more thorough comparison of μCT images between controls and embryos from *Nlrp2*-KO dams (KO × WT matings). We found that, in addition to the anomalies observed by microscopy, embryos from *Nlrp2*-KO dams had abnormal embryonic axes and cardiac deformities (Supplemental Video 3). By examining the μCT data, we were able to visualize embryos within the yolk sac, immersed in amniotic fluid, while still attached to the placentas*.* These findings reflect consistent trends observed across μCT datasets from 29 embryos and align with phenotypes characterized through segmentation analysis. We then performed an in-depth analysis of embryonic anomalies from the μCT dataset using this approach along with μCT 2D sectioning techniques focused on the heart (Supplemental Video 3), and 3D segmentation of the heart using IMARIS software (Figure 4A–D) to systematically evaluate the development and structural defects of the right ventricle, left ventricle, outflow tracts, right atrium, and left atrium. These comprehensive analyses revealed a diverse spectrum of congenital anomalies in embryos derived from KO × WT matings. We found that 31% of embryos of maternal effect (KO × WT) matings had developed normally and were comparable to embryos of control (WT × WT) and paternal effect (WT × KO) matings, but 17% were smaller, 31% had abnormalities in the craniofacial regions, and 13% had an abnormal embryonic axis. Detailed analysis of the heart indicated that 65% had abnormal atria, 62% had abnormal ventricles, and 20% had abnormalities in both atria and ventricles (Figure 4E and F). All embryonic abnormalities, including craniofacial, axial, and cardiac defects, were defined using atlas-based anatomical criteria and μCT-based segmentation, and phenotypic scoring was performed blinded to maternal genotype.

### Maternal loss of *Nlrp2* causes placental abnormalities

**Figure 5:**
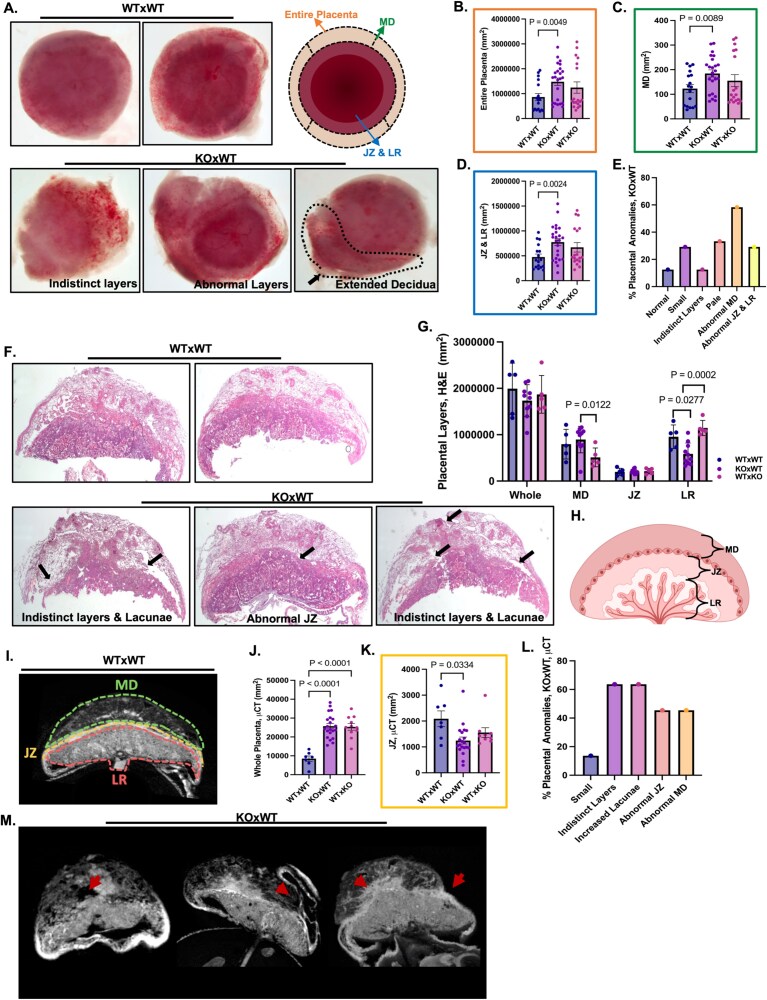
Maternal loss of *Nlrp2* causes placental abnormalities. (A–E) Microscopic examination of E11.5 placentas from KO × WT matings shows variable abnormalities, including reduced overall size, paler appearance, indistinct placental layers, and extended MD, compared to controls (*n* = 24, placentas from independent pregnancies). (B–D) Show quantification of total placental area, MD area, and combined JZ and LR. (E) Shows the relative frequencies of these findings across KO × WT matings. (F–H) H&E-stained cross-sectional area analysis. Placental cross-sections show indistinct layers and increased lacunae (*n* = 10, placentas). Black Arrows denote the sites where decidual and placental anomalies were observed. Quantification of MD, JZ, and LR areas is shown in panel (G). (I–M) μCT analysis of placentas. Red arrows in panel (M) point towards placental anomalies. The entire placental size (J) and JZ (K) are notably affected (*n* = 21, placentas) (Error bars = SEM; Area = mm^2^).

We also performed a comprehensive morphological and histological examination of MD and placental morphology using three complementary methods. First, decidual and placental light microscopy (Figure 5A–E) showed that the decidua and placentas of KO × WT matings have variable abnormalities. Representative images demonstrated that they can be smaller, paler, with indistinct or abnormal layers, and extended MD (Figure 5A). We quantified these changes according to the schematic in Figure 5A. Decidua and placentas from Maternal Effect (KO × WT) matings had significantly differently sized decidua and placental areas compared to WT mating only (*P* = 0.0049; one-way ANOVA with multiple comparisons) (Figure 5B), MD, (*P* = 0.0089; one-way ANOVA with multiple comparisons) (Figure 5C) and combined junctional zone (JZ) and labyrinth (LR) (*P* = 0.0024; one-way ANOVA with multiple comparisons) (Figure 5D). The relative percentages of these findings across all examined placentas from KO × WT matings are in Figure 5E.

Second, H&E staining of cross-sections (Figure 5F–H) revealed that some deciduas and placentas of KO × WT matings had indistinct layer boundaries and an increased number of lacunae across decidua and placental layers (Figure 5F). Quantitative analysis of the layers (Figure 5G) demonstrated no significant differences in area sizes of the JZ and placental cross-sections. However, significant differences were quantifiable in MD (*P* = 0.0122; one-way ANOVA with multiple comparisons) and LR (*P* = 0.0277; *P* = 0.0002; one-way ANOVA with multiple comparisons) (Figure 5G).

Third, we used μCT imaging for a more comprehensive three-dimensional (3D) evaluation of the deciduas and placentas (Figure 5I–M). We extracted three different cross-sections at a 3 μm voxel resolution and standardized sectioning from the μCT imaging datasets, with the placental midpoint serving as the analysis center point. Utilizing 3D-rendering and 2D-sectioning techniques (Supplemental Videos 1–*2*), we found a wide range of anomalies in deciduas and placentas from KO × WT matings *(*Figure 5L and M), confirming the findings from basic microscopy and H&E-stained cross-sections, but at higher resolution and in greater detail. Quantification of MD and placental layer areas was performed based on the schematic in Figure 5I. We found a significant difference in the total area, compared to the WTxWT mating only (*P* < 0.0001; one-way ANOVA with multiple comparisons) (Figure 5J), with a significantly altered JZ, compared to WT mating but not the paternal effect mating (*P* = 0.0334; one-way ANOVA with multiple comparisons) (Figure 5K). There were no significant differences in the MD and LR (Supplemental Figure *2*). All placental abnormalities were defined using anatomical criteria and μCT-based segmentation, and phenotypic scoring was performed blinded to maternal genotype.

### Maternal loss of *Nlrp2* alters transcription in the placenta

**Figure 6:**
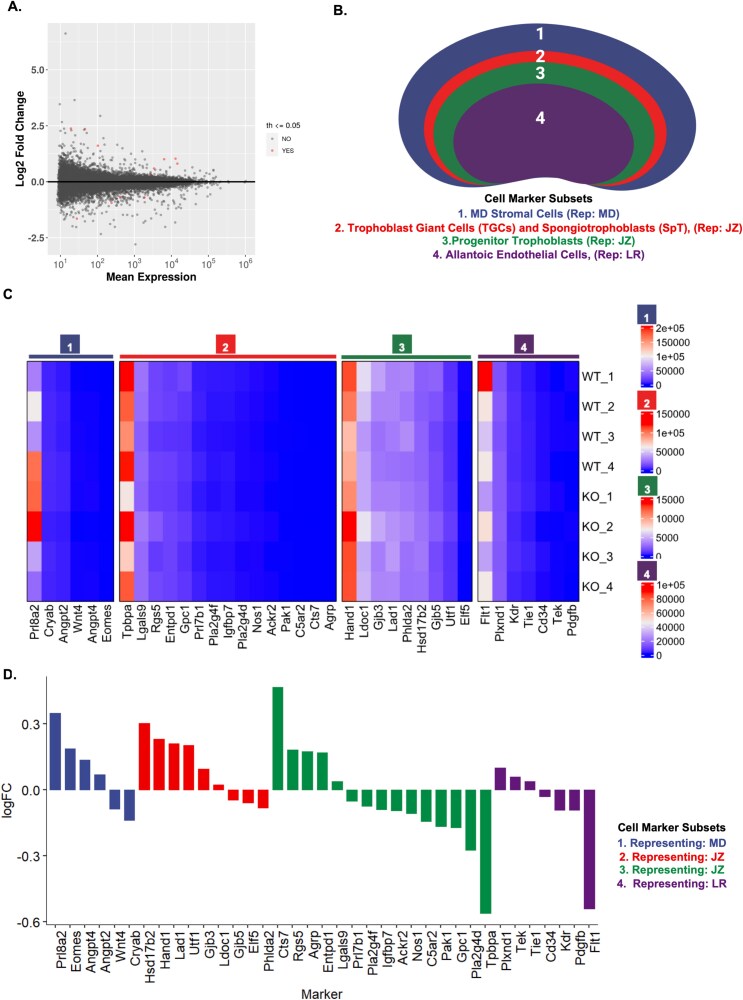
Maternal loss of *Nlrp2* alters transcription in the MD and placenta. (A) MA plot of bulk RNA-seq comparing (WT × WT) and (KO × WT), MD, and placental tissue (*n* = 4 per genotype). Each dot represents a gene (plotted by mean) normalized count (x-axis) and log₂ fold change (y-axis); red dots denote adjusted p < 0.05, |FC| > 1.5. (B) Schematic depiction of four cell marker categories: (1) MD Stromal Cells representing MD; (2) TGCs and Spongiotrophoblasts (SpT) representing the JZ; (3) Progenitor Trophoblasts representing JZ; (4) Allantoic Endothelial Cells representing the LR. (C) Heatmap of log-transformed gene counts for all marker genes in each subset (columns), across individual WT and KO samples (rows). (D) Log₂ fold-change bar graph for each marker gene ordered and colored by subset: MD stromal (blue), TGCs + SpT (red), progenitor trophoblasts (green), and LR endothelial markers (purple). Upward bars indicate genes up-regulated in KO, downward bars indicate down-regulation. Progenitor trophoblast markers (subset 3, green) exhibit the greatest magnitude of dysregulation, consistent with the structural and μCT abnormalities observed in the JZ.

Given the documented morphological anomalies in MD and placental layers from KO × WT matings, we conducted bulk transcriptome profiling using RNA-seq to compare MD and placenta from KO × WT matings to those from control WT × WT matings. Principal component analysis of the RNA-seq data showed minimal separation between samples from the MD and placenta of WT and *Nlrp2*-KO matings (Supplemental Figure 3). This is likely due to the broad range of phenotypic differences arising from maternal loss of *Nlrp2.* As the RNA sequencing was performed on bulk tissue, the results represent an aggregate of multiple placental and decidual cell types. Differentially expressed genes were identified using thresholds of adjusted *P* < 0.05, fold change >1.5, and false discovery rate (FDR) < 0.05 (Figure 6A).

To analyze transcriptome data relative to our decidual and placental histology and μCT findings, we applied a previously established bioinformatic method using the BisqueRNA package [43] to the bulk placental RNA-seq data. This method estimates the relative abundances of various cell types by applying PCA-based decomposition. It utilizes a user-provided list of marker genes to subset the expression data, generating estimates of cell type fractions. These estimates enable us to identify the regions with the most disrupted cell composition resulting from maternal loss of *Nlrp2*. For a more detailed cell type-specific analysis, we categorized cell marker genes into four subsets: (1) Decidual Stromal Cells, corresponding to the MD; (2) Trophoblast Giant Cells (TGCs) and Spongiotrophoblasts (SpT), representing the JZ; (3) Progenitor Trophoblasts, associated with the JZ; and (4) Allantoic Endothelial Cells, representing the LR, where maternal-fetal exchange occurs (Figure 6B, C and Supplemental File 1). Principal component analysis-based decomposition analysis of these four distinct maternal decidual and placental cell marker subsets revealed that subset progenitor trophoblasts (subset 3), associated with the trophoblast progenitor compartment and JZ, exhibited the most transcriptional variability in maternal *Nlrp2*-KO placentas (Figure 6B and D). This marked shift in progenitor marker expression suggests a disruption in trophoblast lineage specification and maintenance. These transcriptomic findings align with our histological and μCT analyses, which also demonstrated structural disorganization and increased variability specifically in and around the JZ. Together, these data indicate that the JZ and its progenitor cell populations are likely disrupted by the maternal *Nlrp2* loss, implicating this layer as a key site of placental dysfunction.

**Figure 7:**
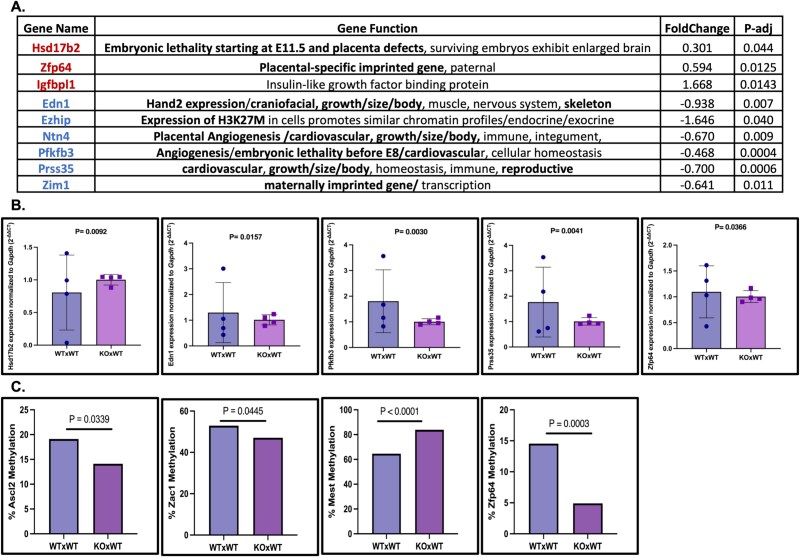
Validation of placental RNA-seq data and assessment of imprinting-associated alterations. (A) List of nine selected developmentally important genes from the DEG list identified by RNA-seq. (B) RT-qPCR for these genes using *Gapdh* as a reference housekeeping gene. Differential expression was confirmed for *Hsd17B2*, *Edn1*, *Pfkfb3*, *Prss35, and Zfp64* in KO × WT placentas compared to WT × WT. (Error bars = SEM). (C) Bisulfite sequencing of four imprinting-associated regions, *Zac1, Mest, Ascl2, Zfp64*, in placental tissue. Bar plots show mean percent DNA methylation per placenta for WT × WT and KO × WT samples, demonstrating locus-specific methylation alterations associated with maternal loss of *Nlrp2* (*n* = 4 placentas per group).

Based on the literature review, we next selected nine developmentally significant genes from the DEGs adjusted *P* < =0.05, FC > 1.5, FDR < 0.05 to validate the RNA-seq results by RT-qPCR using *Gapdh* as a control (Figure 7A, B and Supplemental File 2*).* Five of these genes, *Hsd17B2*, *Edn1*, *Pfkfb3*, *Prss35*, and *Zfp64,* were confirmed to be differentially expressed in placentas from KO × WT matings compared to WT × WT matings (Figure 7B, Supplemental Figure 5 and Supplemental File 2*)*. We also noted that the expression of these five validated genes is more variable in the placentas of control WT × WT matings compared to those of KO × WT matings. This might be attributed to the inherent genetic and regulatory complexity present in WT organisms [47]. The expression by RT-qPCR of four other tested genes (*Igfbpl1*, *Ezhip*, *Ntn4*, and *Zim1*) was comparable between genotypes (Supplemental Figure 5 and Supplemental File 2).

Our study next focused on identifying candidate marker genes linked to both cardiac and placental development, leveraging the “placenta-heart axis” concept [34, 35, 48]. A curated list of seven genes, *Gata4, Hand1, Hand2, Tbx5, Tbx20, Isl1, and Gja5* (Supplemental Figure 4A), was derived through extensive literature supporting evidence for CHDs and placental vascular development [32–35]. These genes are critical for processes such as septation, chamber formation, and outflow tract development in the heart, and several also contribute to endothelial or trophoblast function within the placenta [33–35, 48]. Several of these placental–cardiac marker genes exhibited distinct expression patterns between *Nlrp2*-KO and WT samples during virtual cell decomposition (Supplemental Figure 4B). It is important to note that this analysis also utilized the BisqueRNA package and aimed to estimate the relative abundances of cell types from PCA-based decomposition using a user-provided gene list. Although our methodology was based on an extensive literature review, additional experimental validation is needed to confirm these findings (Supplemental Figure 4).

### Maternal loss of *Nlrp2* alters DNA methylation at selected imprinting-associated loci in the placenta

We next evaluated DNA methylation at selected imprinting-associated loci in placental tissue from *Nlrp2* (KO × WT) and (WT × WT) matings. Bisulfite sequencing of four loci that passed quality control (*Ascl2*, *Zac1*, *Mest*, and *Zfp64*) revealed consistent locus-specific alterations in placenta-level mean DNA methylation associated with maternal loss of *Nlrp2*. Placental tissue from KO × WT matings showed significant hypomethylation at *Ascl2* (*P* = 0.0339), *Zac1* (*P* = 0.0445), and *Zfp64* (*P* = 0.0003), and significant hypermethylation at *Mest* (*P* < 0.0001) based on comparisons of mean percent methylation per placenta, compared to WT controls (Figure 7C). These changes were evident both in the averaged methylation percentages and in the CpG-by-CpG distribution profiles (Supplemental Figure 6 and Supplemental File 4). Together, these results demonstrate that maternal loss of *Nlrp2* leads to locus-specific methylation abnormalities in placental tissue, supporting a role for maternal *Nlrp2* in imprinting reprogramming.

### Clinical studies and human *NLRP2* variant analysis.

**Figure 8:**
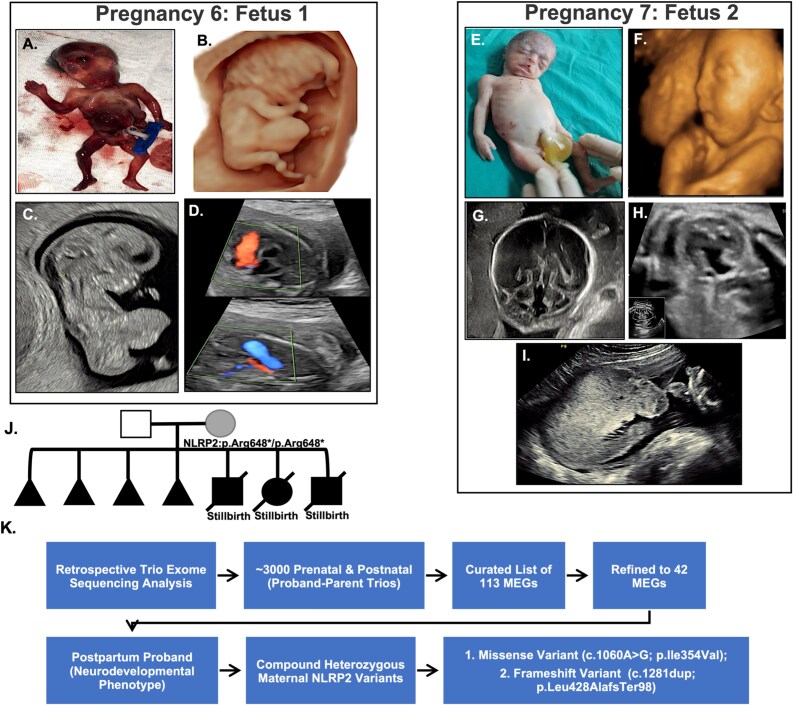
Prenatal, postnatal, and genetic findings in two mother-offspring dyads revealing maternal NLRP2 variants. Pregnancy 6 (18 weeks’ gestation; Fetus 1): A–D. (A–B) Postmortem image and 3D ultrasound surface rendering of the fetus show a flat facial profile and a large anterior abdominal wall defect (omphalocele). (C) First-trimester sagittal ultrasound (13 weeks) reveals increased nuchal translucency and a visible omphalocele. (D) Four-chamber and color Doppler fetal echocardiography views demonstrate unidirectional flow through the right ventricle, consistent with hypoplastic left heart syndrome (HLHS). Pregnancy 7 (28–32 weeks’ gestation; Fetus 2): E–I. (E) Postmortem photograph and 3D ultrasound surface rendering of the fetus show craniofacial anomalies, including midface hypoplasia and a prominent omphalocele. (F–G) Axial transcerebellar ultrasound view shows bilateral ventriculomegaly, cerebellar hypoplasia, vermian agenesis, and an open fourth ventricle communicating with an enlarged cisterna magna. (H) Coronal ultrasound image shows a hypoplastic right kidney with a dilated pelvicalyceal system. (I) Transverse view of the uterus showing a markedly enlarged fundal placenta with abnormal morphology. (J) Family pedigree using standard symbols, with affected individuals shaded in black, intrauterine fetal death (IUFD). (K) Genome-wide trio exome analysis identifies compound heterozygous maternal NLRP2 variants. Study design schematic showing retrospective trio-exome sequencing analysis of approximately 3000 prenatal and postnatal proband–parent trios. A curated list of 113 MEGs was refined to 42 genes based on prior associations with structural anomalies. A postpartum proband with a neurodevelopmental phenotype linked to compound heterozygous NLRP2 variants in the mother: a likely pathogenic frameshift variant (c.1281dup; p. Leu428AlafsTer98) and a missense variant (c.1060A > G; p. Ile354Val).

Human bi-allelic maternal *NLRP2* pathogenic variants are associated with recurrent pregnancy loss and children with multilocus imprinting disorders [2, 4–6, 19], but their association with other pregnancy and offspring phenotypes, including different structural anomalies, is unknown. We therefore searched clinic and laboratory data for women with combinations of recurrent pregnancy loss and fetuses or children with congenital anomalies not otherwise explained through genetic testing, which includes proband or trio genome-wide sequencing. We identified two interesting mother-offspring dyads. The first presented in her seventh pregnancy with a history of six prior pregnancies with no term deliveries or surviving children. Two had delivered preterm, two were first-trimester losses, and two were pregnancy losses at 18 and 21 weeks with fetuses with documented structural malformations (Figure 8). There were multiple anomalies, fetal growth restriction, and an enlarged placenta with abnormal vascularity in the index pregnancy, which resulted in stillbirth at 32 weeks. Fetal anomalies included craniofacial malformations, hypoplastic left heart syndrome, omphalocele, bilateral ventriculomegaly, agenesis of the corpus callosum, cerebellar hypoplasia, and hypoplastic right kidney with pelvicalyceal dilation (Figure 8; Table 1). Exome sequencing (ES) on DNA extracted from maternal and paternal blood identified a maternal homozygous nonsense variant in *NLRP2* (NM_017852.5: c.1942C > T, p.Arg648*) that introduces a premature stop codon in exon 6 of 13, classified as likely pathogenic by ACMG criteria [45]. The father did not carry the variant, and neither parent had other variants relevant to the fetal phenotype, such as shared carrier status for pathogenic variants in recessive disease genes. Several of the findings, fetal growth restriction, craniofacial malformations, CHDs, and enlarged placenta with abnormal vascularity in the index pregnancy, are comparable to the phenotypes in embryos of *Nlrp2*-KO female mice.

**Table 1:** Comparison of phenotypes observed in mouse concepti and case 1 with human maternal pathogenic NLRP2 variant. Mouse embryos from maternal *Nlrp2-*KO dams and human homozygous maternal NLRP2 truncating variant cases exhibit overlapping defects that affect embryonic viability, craniofacial and cardiac development, extraembryonic tissues, and neurodevelopment, supporting a conserved maternal-effect mechanism

**Phenotypic feature in pregnancy and offspring**	**Mouse: maternal Nlrp2-KO**	Human: maternal homozygous NLRP2: c.1942C > T (p.Arg648^*^)
[Embryo viability]	Increased resorption; reduced litter size	Recurrent pregnancy loss
**Craniofacial anomalies**	Midface hypoplasia; facial asymmetry	Flat facial profile (Fetus 1) Midface hypoplasia, dysmorphic features (Fetus 2)
**Abdominal wall defects**	Occasional body-wall abnormalities	Omphalocele (Fetus 1 & 2)
**Cardiac defects**	Abnormal cardiac morphology (atrial, ventricular, and outflow tract defects)	Hypoplastic left heart syndrome (Fetus 1)
**Yolk sac/extraembryonic anomalies**	Thinned or absent vitelline vessels; altered yolk sac–embryo ratio	Cannot evaluate
**Placental morphology**	Variable size; indistinct layer organization; expanded lacunae	Enlarged, morphologically abnormal placenta (Fetus 2)
**Neurodevelopmental anomalies**	Neural tube patterning defect; abnormalities in forebrain, midbrain, and overall brain morphogenesis	Bilateral ventriculomegaly, cerebellar hypoplasia, vermian hypoplasia, enlarged cisterna magna (Fetus 2)
**Other findings**	Abnormal embryonic axes; craniofacial disorganization; irregular neural tube architecture; overall embryonic asymmetry	Increased nuchal translucency (Fetus 1) Hypoplastic right kidney with dilated pelvicalyceal system (Fetus 2)

To find the second dyad, we retrospectively searched annotated variants in a genome-wide trio-exome dataset from approximately 3000 prenatal and postnatal probands at Baylor Genetics for variants in a curated list of 113 MEGs, of which 42 were prioritized based on their known roles in early development and otherwise unexplained congenital anomalies or severe developmental phenotypes (Supplemental File 6). This search strategy (Figure 8K) revealed *NLRP2* as the second most frequently annotated gene. We found compound heterozygous variants in *NLRP2* in the mother (but not in the father) of a child presenting with generalized epilepsy with myoclonic jerks, speech delay, and a life-threatening episode of apnea and pneumonia. No other variants were identified in the parents or proband that could explain the phenotype. The first variant was likely pathogenic (ACMG criteria PVS1, PM2) frameshift variant in exon 5, NM_017852.5: c.1281dup (NP_060322.1: p. (Leu428AlafsTer98)), predicted to result in nonsense-mediated mRNA decay and loss of function. The second, a missense variant NM_017852.5: c.1060A > G (NP_060322.1: p. (Ile354Val)), with low in silico pathogenicity prediction (CADD = 0.001, REVEL = 0.179, GERP = −3.09) and present in gnomAD (0.005026), was reported as benign/likely benign in ClinVar. However, this variant was previously described in a heterozygous state in a mother of a patient with pseudohypoparathyroidism type 1B, an imprinting disorder [49]. Although the maternal *NLRP2* genotype in this family is intriguing, the relevance to the child’s phenotype will need further validation (Figure 8K). Nevertheless, the clinical findings, particularly in case 1, support the hypothesis that maternal NLRP2 dysfunction can contribute to a broad spectrum of fetal and placental developmental phenotypes (Table 1) and that analyzing the maternal genome in trio exomes for variants in this or other MEGs could be considered when exome or genome results of those affected probands are non-diagnostic. Detailed clinical data and results information are provided in the Supplemental File 5.

## Discussion

To address the hypothesis that maternal *Nlrp2* loss in mice leads to a wide spectrum of embryonic developmental anomalies that parallel anomalies found in pregnancies and offspring of women with pathogenic variants in *NLRP2*, we first used enhanced imaging methods to characterize a previously uncharacterized broad spectrum of congenital anomalies and disrupted development and vascularization of placentas and yolk sacs from pregnancies of *Nlrp2*-KO female mice. These were associated with transcriptome changes and methylation differences at selected imprinting-associated loci in the placentas of *Nlrp2-KO* females. We next identified two human females with maternal variants in *NLRP2* and described phenotypes in pregnancies and offspring that were not previously reported [8]. The partial phenotypic overlap with findings in the mice provides support for the hypothesis that human MEG mutations can cause structural anomalies and other developmental phenotypes, in addition to previously described multilocus imprinting disorders [5, 6]. These data provide new insights into the role of maternal NLRP2 in both embryonic and extra-embryonic development in mice and humans.

The use of advanced microscopy and 3D μCT imaging allowed us to examine phenotypes more efficiently and at high resolution in embryos, yolk sacs, and placentas as a unit. This was critical to uncovering the abnormally thin or missing vitelline arteries in the yolk sac vasculature, reduced embryo to yolk sac area ratios, and increased embryo resorption in concepti from *Nlrp2*-KO dams, supporting that maternal NLRP2 is important for the early development of the embryo and placenta and that its presence impacts expression of genes coding for angiogenic factors or other factors required for vasculogenesis and angiogenesis [50, 51]. The broadened spectrum of congenital anomalies in embryos from *Nlrp2*-KO dams includes craniofacial, embryonic axis, and cardiac malformations. This reinforces the link between maternal loss-of-function of *Nlrp2* and a wide array of different congenital anomalies. We focused deeper on CHDs because they are among the most common and complex anomalies in humans, and in many cases, the etiology remains unknown even after comprehensive diagnostic workups, and a previous report suggested possible contributions of MEG variants [33, 34, 52, 53]. The dual impact of a MEG on the development of both the embryonic heart and the placenta is supported by previous evidence that cardiac development is closely linked to placental function and structure during embryogenesis [34, 35]. Specifically, the placenta provides essential signals and nutrients necessary for proper heart development, and alterations in placental development can lead to insufficient support for embryonic cardiac structures [33–35, 48]. Our findings extend the “placenta-heart axis” concept by showing that maternal *Nlrp2* loss coincides with abnormalities in both placental and cardiac development, supporting further investigation of MEGs in CHDs [7, 52].

The high-resolution analysis of placental morphology and transcriptomics revealed notable variability in placental size with indistinct layers and increased lacunae, along with notable disruptions in the JZ of placentas from *Nlrp2*-KO dams. Using PCA-based decomposition on bulk placental RNA, we also identified significant variations in the cell type-specific composition within the placental tissue, which was also most prominent in the JZ region. This convergence of morphological, μCT, and transcriptomic data strongly suggests that the JZ is a critical site of vulnerability to maternal *Nlrp2* loss. Finally, the locus-specific alterations in DNA methylation at four imprinting-associated regions in the placenta was consistent with our prior data showing altered imprinting in *Nlrp2*-deficient embryos [13, 17]. We now extend those observations to the extra-embryonic compartment, providing further evidence that disrupted maternal epigenetic programming contributes to the developmental abnormalities observed.

The two human mother-offspring dyads support the translational relevance of the mouse phenotypes. In particular, the recurrent adverse outcomes and fetal and placental phenotypes in patients with homozygous maternal *NLRP2* pathogenic variants are comparable to those observed in the *Nlrp2*-KO mouse. The second case, involving compound heterozygous maternal NLRP2 variants and a child with a neurodevelopmental phenotype, may represent a potential phenotype expansion but is hypothesis-generating and requires independent confirmation, as the clinical relevance of one variant remains uncertain despite prior reporting in a parent of a child with MLID [49]. Nevertheless, these cases support the clinical utility of evaluating maternal genotypes in selected families with unexplained structural anomalies or pregnancy loss after standard genomic testing.

The observed wide array of mid-gestation anomalies indicates that *Nlrp2*, which, like other SCMC-encoding MEGs, is expressed during oogenesis and early embryogenesis, has distinct downstream effects—beyond preimplantation development—on the development of different organs and extra-embryonic tissues. Our previous data [21, 25] and that of studies on other SCMC genes [10, 14] indicate that by regulating processes such as ZGA and embryonic axis formation, maternal *Nlrp2* can influence both embryonic and extra-embryonic development, ultimately impacting organogenesis and placental morphogenesis [7, 13, 14, 17, 54]. Whether these defects arise solely from oocyte-driven mechanisms or whether the maternal reproductive tract environment also contributes remains incompletely addressed, but we note that in our previous experiments, transfer of mouse embryos derived from *Nlrp2*-KO oocytes into WT uteri did not rescue the altered reproductive outcomes and embryonic developmental defects [17].

In conclusion, by integrating animal models, transcriptomics, and clinical genetics, we demonstrate that maternal NLRP2 dysfunction is associated with a spectrum of anomalies ranging from pregnancy loss to offspring with multiple congenital anomalies. Our findings support the central hypothesis that maternal NLRP2 plays an important role in normal embryonic and placental development. They further support the importance of including MEGs such as NLRP2 in reproductive genetic evaluation and counseling.

## Supplementary Material

Supplementary_materials_Video_1_ioaf290

Supplementary_materials_Video_2_ioaf290

Supplementary_materials_Video_3_ioaf290

Supplementary_materials_Figure_1_ioaf290

Supplementary_materials_Figure_2_ioaf290

Supplementary_materials_Figure_3_ioaf290

Supplementary_materials_Figure_4_ioaf290

Supplementary_materials_Figure_5_ioaf290

Supplementary_materials_Figure_6_ioaf290

Supplementary_materials_File_1_ioaf290

Supplementary_materials_File_2_ioaf290

Supplementary_materials_File_3_ioaf290

Supplementary_materials_File_4_ioaf290

Supplementary_materials_File_5_ioaf290

Supplementary_materials_File_6_ioaf290

## Data Availability

The placental RNA sequencing data have been deposited in GEO under accession number: GSE274909.
