# Supplementary figures and images for: Loss of the maternal effect gene NLRP2 impairs embryonic and extra-embryonic development, revealing a novel genetic cause of congenital anomalies
Source: Biol Reprod. 2025 Dec 27;114(4):1469–85. doi: 10.1093/biolre/ioaf290 (PMC13079454; doi:10.1093/biolre/ioaf290)

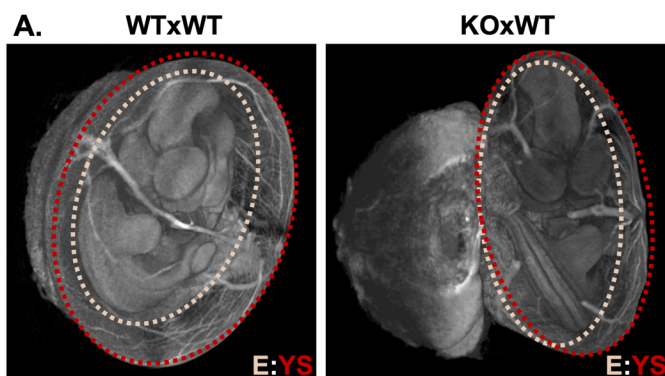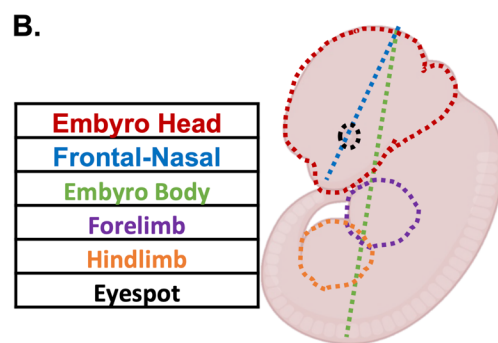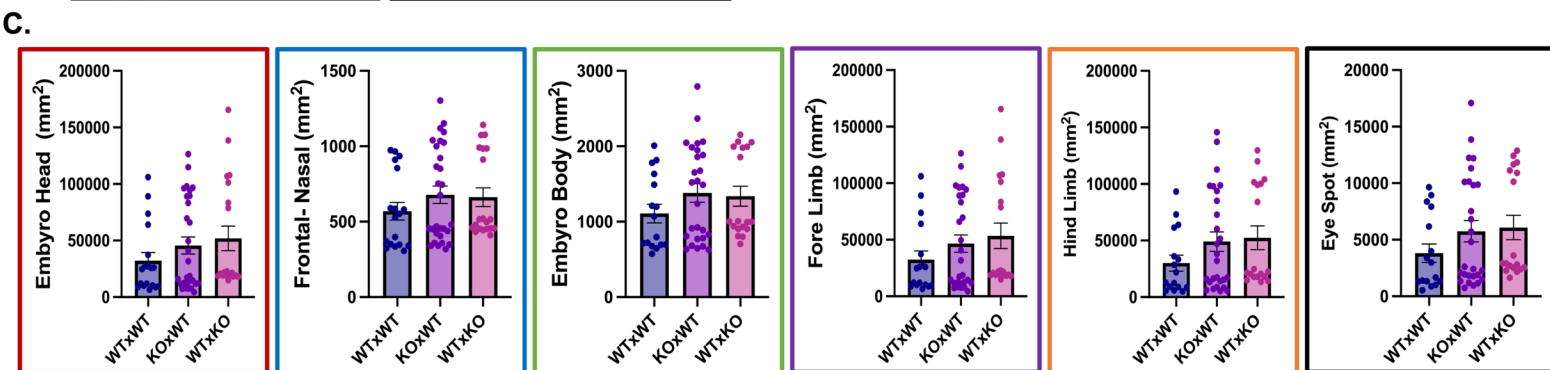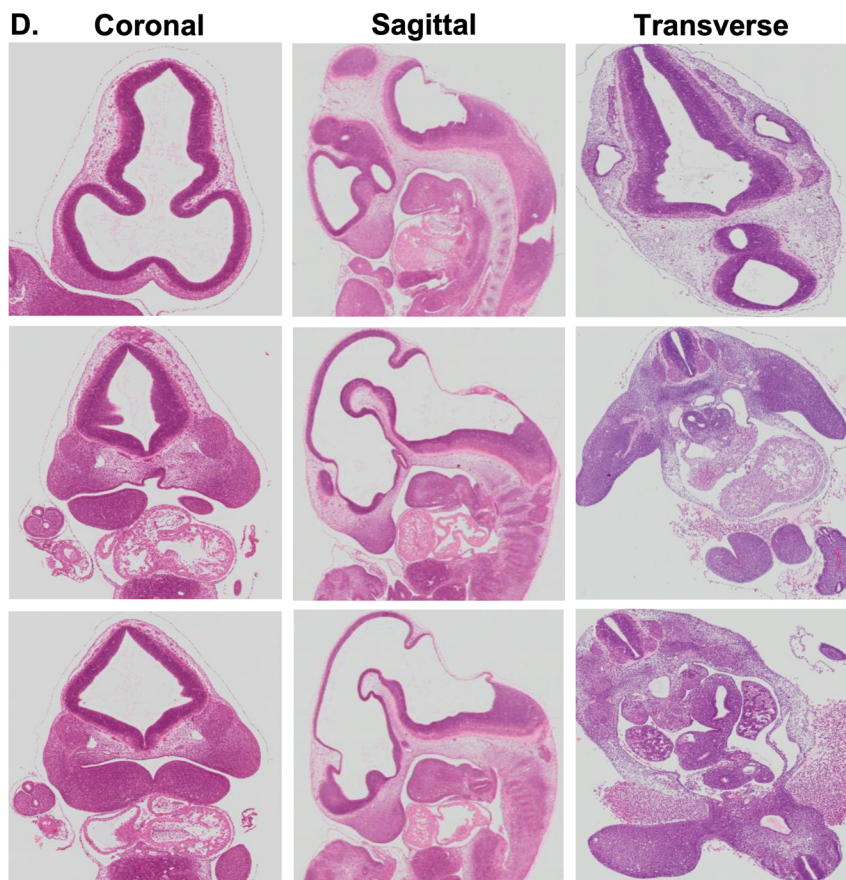

Supplement: Supplementary_materials_Figure_1_ioaf290 [file supplementary_materials_figure_1_ioaf290.pdf]

**A.**

PCA 12

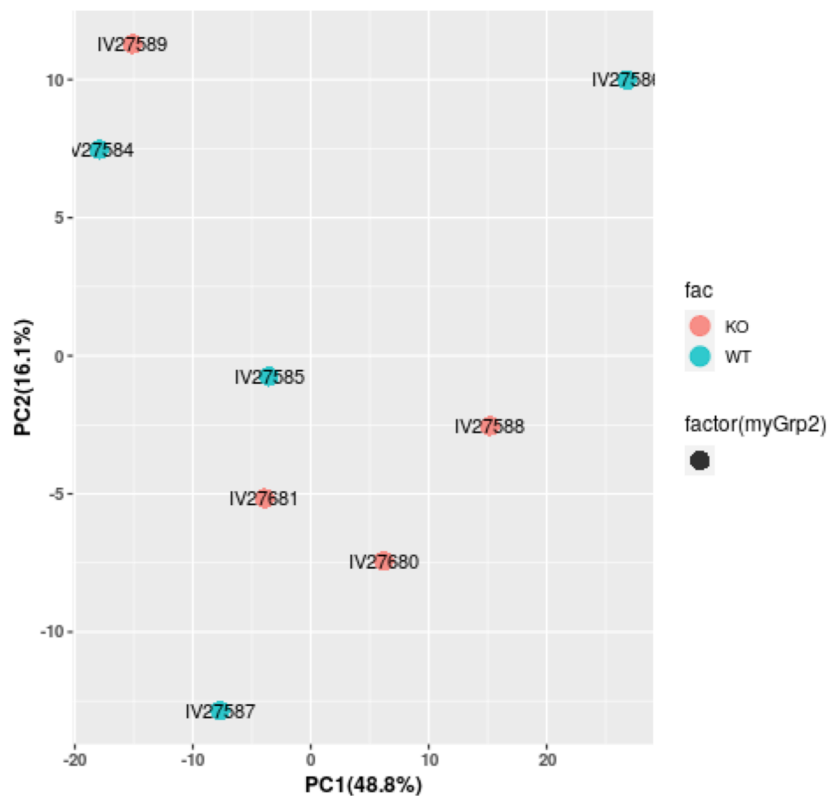

Supplement: Supplementary_materials_Figure_2_ioaf290 [file supplementary_materials_figure_2_ioaf290.pdf]

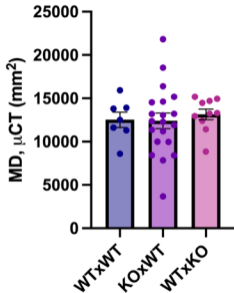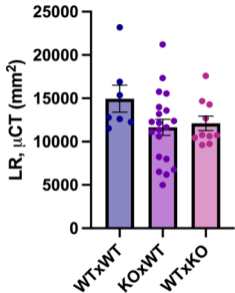

Supplement: Supplementary_materials_Figure_3_ioaf290 [file supplementary_materials_figure_3_ioaf290.pdf]

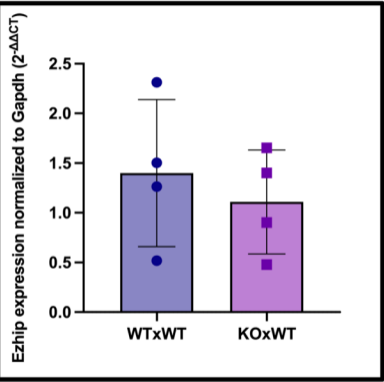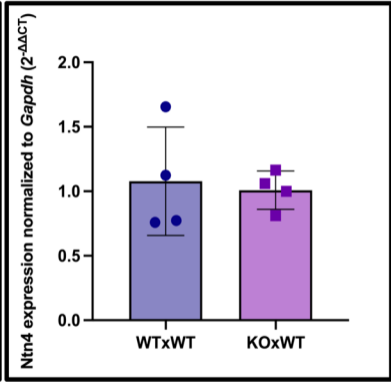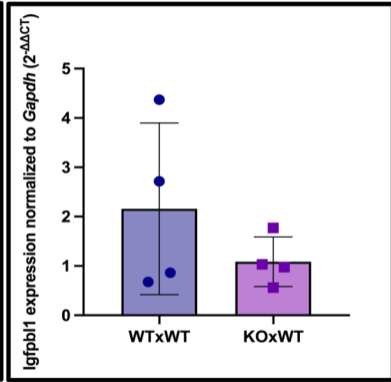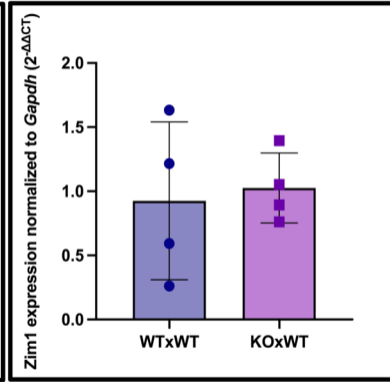

Supplement: Supplementary_materials_Figure_5_ioaf290 [file supplementary_materials_figure_5_ioaf290.pdf]

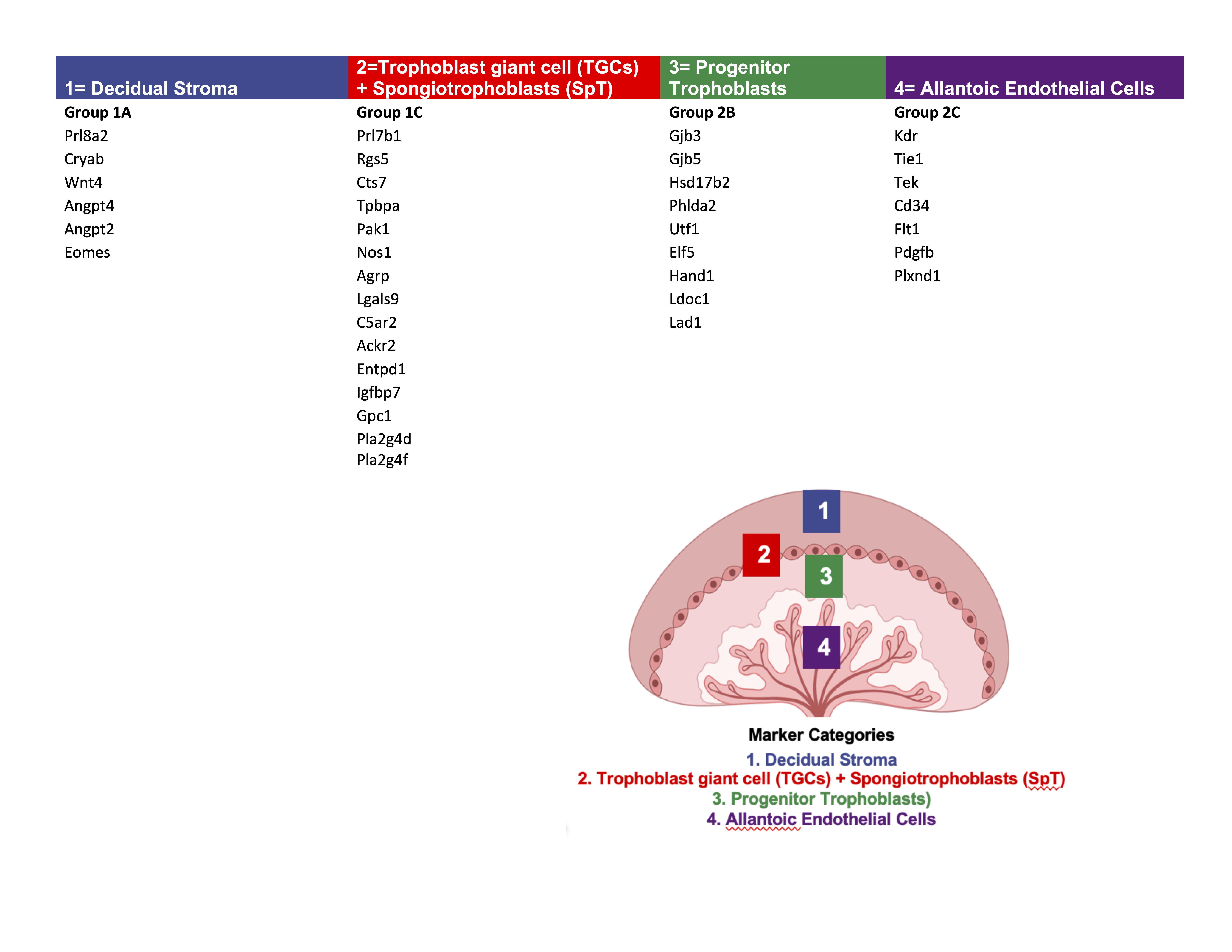

Supplement: Supplementary_materials_File_1_ioaf290 [file supplementary_materials_file_1_ioaf290.jpeg]
