# Supplementary material for: Loss of the maternal effect gene NLRP2 impairs embryonic and extra-embryonic development, revealing a novel genetic cause of congenital anomalies
Source: Biol Reprod. 2025 Dec 27;114(4):1469–85. doi: 10.1093/biolre/ioaf290 (PMC13079454; doi:10.1093/biolre/ioaf290)

A.

**Gata4:** Mutations in the Gata4 gene have been linked to CHD, particularly in the context of atrial septal defects and ventricular septal defects.
**Gja5:** Gja5 is expressed in the endothelial cells of the fetal vessels. Its expression is critical for proper vascular development and function.
**Hand1:** Mutations in the Hand1 gene have been found to cause cardiac defects, including abnormal development of the heart chambers and valves.
**Hand2:** Mutations in the Hand2 gene are associated with CHD, such as ventricular septal defects and outflow tract abnormalities.
**Isl1:** Mutations in the Isl1 gene have been linked to CHD, including double outlet right ventricle (DORV) and atrioventricular septal defects (AVSDs).
**Tbx5:** Mutations in the Tbx5 gene are associated with CHD, including Holt-Oram syndrome, which manifests as heart defects along with limb abnormalities.
**Tbx20:** Mutations in the Tbx20 gene have been identified in mice with CHD, particularly affecting the outflow tract and ventricular septum development.

B.

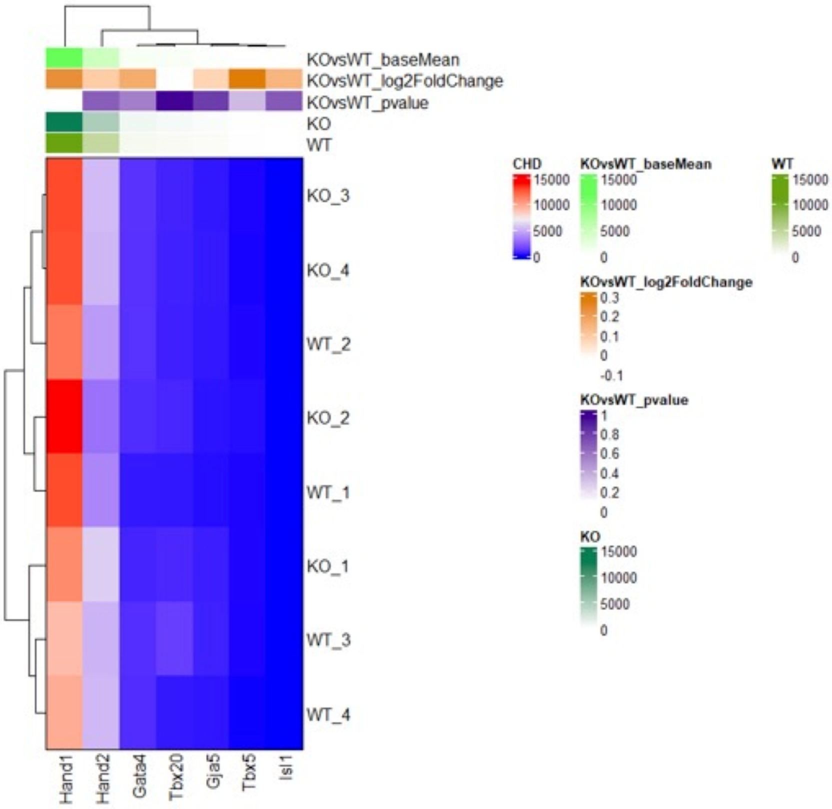

Supplement: Supplementary_materials_Figure_4_ioaf290 [file supplementary_materials_figure_4_ioaf290.pdf]
