# Supplementary material for: Loss of the maternal effect gene NLRP2 impairs embryonic and extra-embryonic development, revealing a novel genetic cause of congenital anomalies
Source: Biol Reprod. 2025 Dec 27;114(4):1469–85. doi: 10.1093/biolre/ioaf290 (PMC13079454; doi:10.1093/biolre/ioaf290)
Supplement: Supplementary_materials_Figure_6_ioaf290 [file supplementary_materials_figure_6_ioaf290.pdf]

A.

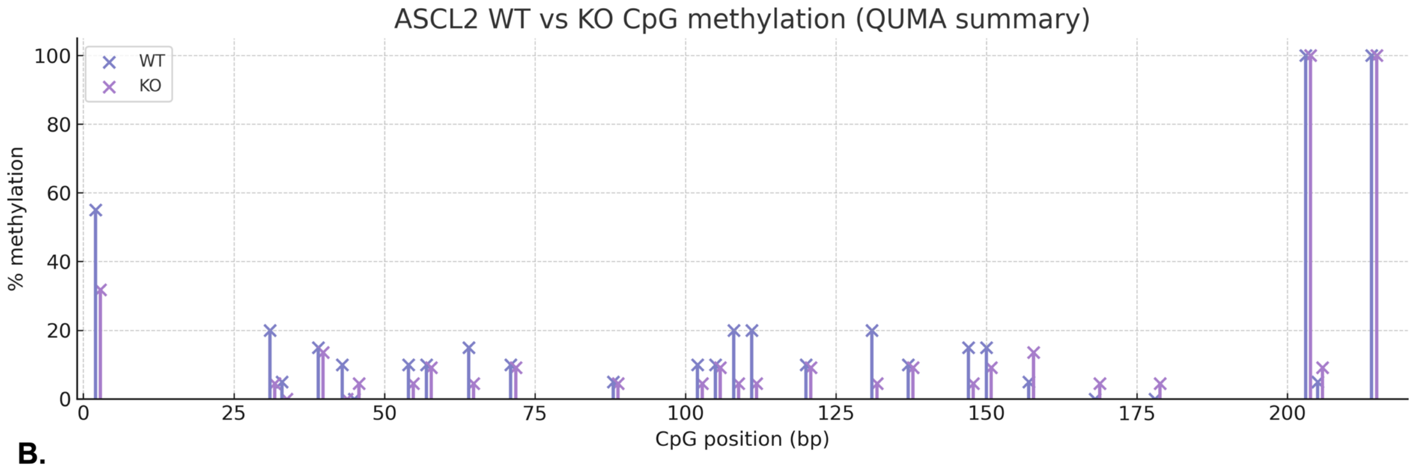
ASCL2 WT vs KO CpG methylation (QUMA summary)
% methylation
CpG position (bp)
WT
KO

B.

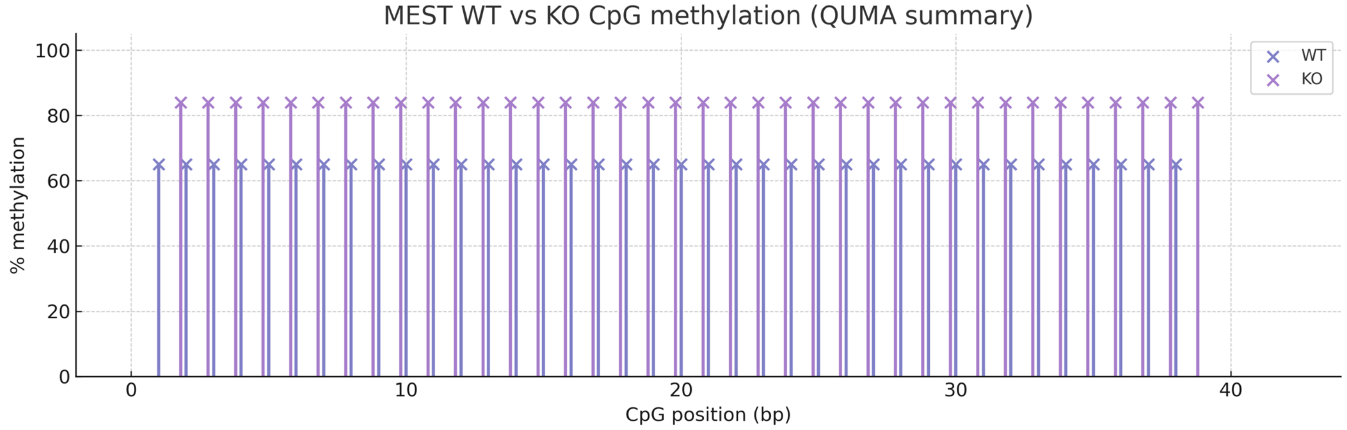
MEST WT vs KO CpG methylation (QUMA summary)
% methylation
CpG position (bp)
WT
KO

C.

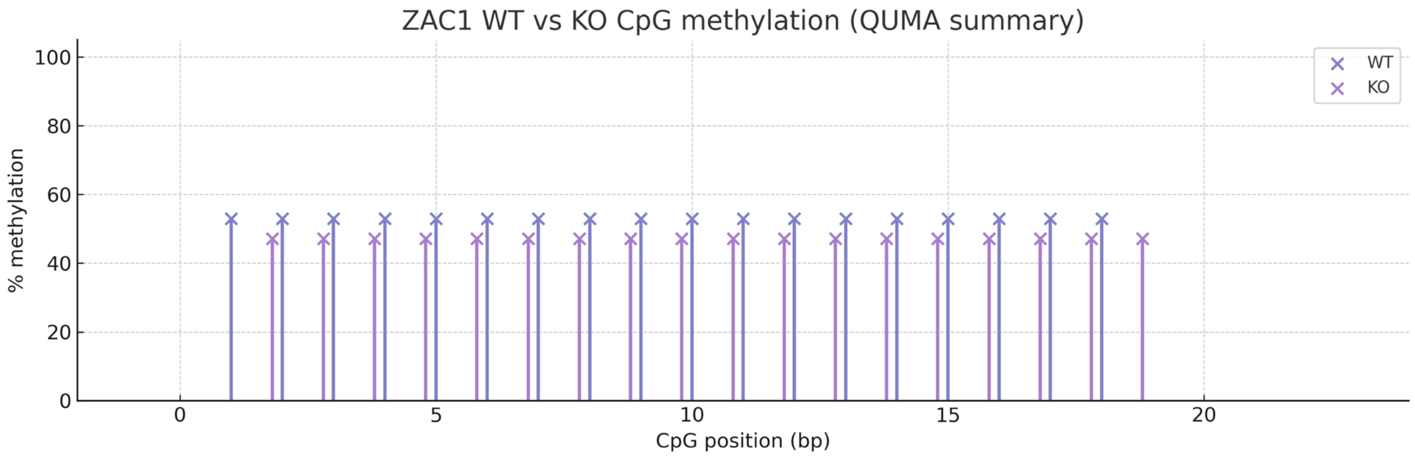
ZAC1 WT vs KO CpG methylation (QUMA summary)
% methylation
CpG position (bp)
WT
KO

D.

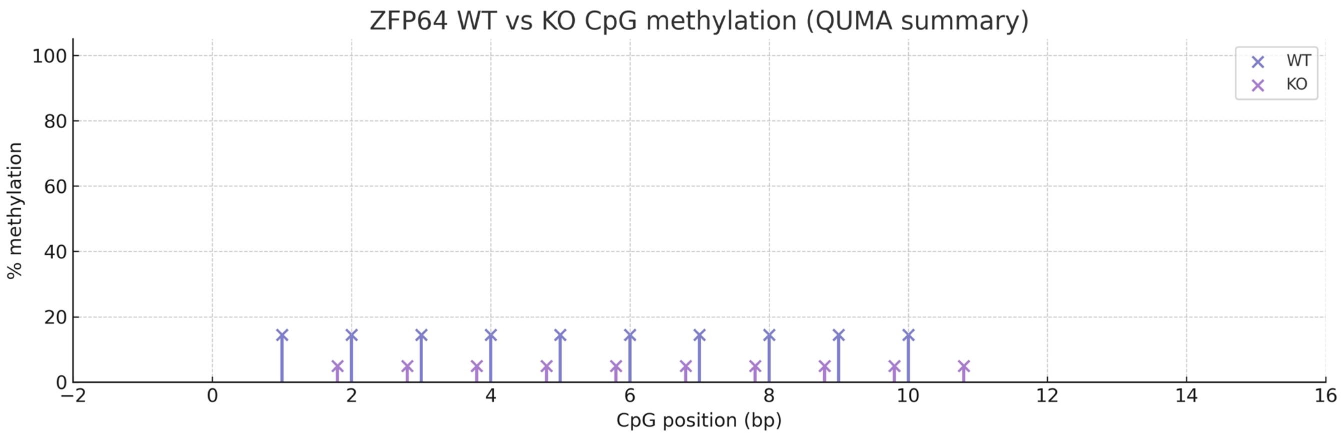
ZFP64 WT vs KO CpG methylation (QUMA summary)
% methylation
CpG position (bp)
WT
KO
