# Supplementary material for: Loss of the maternal effect gene NLRP2 impairs embryonic and extra-embryonic development, revealing a novel genetic cause of congenital anomalies
Source: Biol Reprod. 2025 Dec 27;114(4):1469–85. doi: 10.1093/biolre/ioaf290 (PMC13079454; doi:10.1093/biolre/ioaf290)
Supplement: Supplementary_materials_File_5_ioaf290 [file supplementary_materials_file_5_ioaf290.docx]

**Supplemental Clinical Case Results**

**Detailed clinical information on the first human mother-offspring dyad:**

The patient was a 31-year-old gravida 7 with an obstetric history of no term and 2 preterm deliveries, 4 spontaneous pregnancy losses (2 in the first trimester and one each at 18 and 21 weeks), and no living children (G7-0-2-4-0). The two second-trimester pregnancy losses both involved fetuses with documented structural malformations (Fetus 1 in Fig. 8). She was referred at 28 weeks’ gestation for prenatal genetic consultation because of multiple fetal anomalies detected by a detailed ultrasound anomaly scan that showed fetal growth restriction (all measurements <5th percentile), enlarged placenta with abnormal vascularity, craniofacial malformations (midface hypoplasia, hypertelorism, micrognathia, and low-set ears), hypoplastic left heart syndrome (confirmed by fetal echocardiography), omphalocele containing bowel loops, bilateral ventriculomegaly, agenesis of the corpus callosum, cerebellar hypoplasia, and hypoplastic right kidney with pelvicalyceal dilation (Fetus 2 in Fig. 8). At 32 weeks’ gestation, the patient experienced preterm labor and delivered a stillborn male fetus. An external postmortem examination confirmed the major anomalies identified on prenatal ultrasound, including the omphalocele and dysmorphic facial features, but a full fetal autopsy was declined. The clinical findings in this stillborn fetus are comparable to key phenotypes observed in embryos of *Nlrp2*-KO mouse models, specifically fetal growth restriction, craniofacial malformations, and CHDs. Moreover, the enlarged placenta with abnormal vascularity is reminiscent of the placental morphological and vascular defects identified in the maternal KO mouse concepti.

**Clinical information on the second human mother-offspring dyad:**

Trio exome sequencing was done for a child presenting with a neurodevelopmental phenotype. The proband exhibited generalized epilepsy with myoclonic jerks, speech delay, and a life-threatening episode involving apnea and pneumonia. Despite extensive clinical and genetic evaluation, including trio exome sequencing, of the proband, no genetic cause was identified that could explain the phenotype. No additional phenotype information was available.
